# Editorial: Advanced Membrane Science and Technology for Sustainable Environmental Applications

**DOI:** 10.3389/fchem.2020.609774

**Published:** 2020-11-12

**Authors:** Shuaifei Zhao, Liguo Shen

**Affiliations:** ^1^Institute for Frontier Materials, Deakin University, Geelong, VIC, Australia; ^2^College of Geography and Environmental Sciences, Zhejiang Normal University, Jinhua, China

**Keywords:** membrane separation, desalination, water treatment, gas separate membranes, carbon capture

Over the past few decades, membrane technologies have attracted growing interest and applications in various fields due to their high separation efficiency, cost-effectiveness, modularity, and low footprint (Yan et al., 2015; Thomas et al., 2017). In particular, membrane separation has been widely used for liquid and gas separations, most of which are related to environmental challenges. Numerous membrane materials and processes have been investigated to address environmental concerns, such as adsorptive membranes for pollutant removal (Zhang et al., 2018), catalytic membranes for organic degradation (Li et al., 2020), gas separation membranes for carbon capture (Song et al., 2016), membrane condensation for resource recovery (Yue et al., 2016), and membrane evaporation for energy savings (Zhao et al., 2015a,b).

For liquid separation (e.g., desalination and wastewater treatment), there many challenges in developing membranes applications, such as membrane fouling (Liu et al., 2018), low chemical stability (Wang et al., 2018), and low water permeability (Wang et al., 2019). Xu et al. reviewed the roles of membrane-foulant and foulant-foulant intermolecular interactions during organic fouling of microfiltration (MF) and ultrafiltration (UF) membranes. They also summarized the organic fouling mechanisms, including non-covalent interactions (e.g., electrostatic interactions and hydrophobic interactions based on van der Waals and Lewis acid-base interactions), covalent interactions (e.g., metal-organic complexation), and spatial effects. According to the mechanisms at different fouling stages, various antifouling strategies, such as hydrodynamic control, membrane modification, foulant conditioning, and membrane cleaning were proposed. Shen et al. summarized the membrane antifouling modification methods using ZnO nanoparticles, including internal (bulk) modification and external (surface) modification. Several types of membranes, such as polyvinylidene fluoride (PVDF)-ZnO, polyethersulfone (PES)-ZnO, and other polymers-ZnO composite membranes were briefly reviewed. The conclusions from these two review papers are applied to porous (e.g., MF and UF) membranes and they may not be applicable for dense (e.g., reverse osmosis) membranes.

As an emerging membrane technology, forward osmosis (FO) has been intensively investigated for desalination (Zhao et al., 2012). However, the desalination performance of FO membranes in terms of water permeability, salt selectivity, and concentration polarization still needs improvement (Kahrizi et al., 2020). Li et al. reported thin film composite FO membranes with graphene oxide nanosheets incorporated in the polyamide layer during interfacial polymerization. The FO membranes showed higher water flux, lower reverse solute diffusion, lower structural parameters, and higher chlorine resistance compared with the control membrane. Mei et al. reported ZIF-8/polysulfone-mixed matrix membranes with improved selectivity for H_2_/CO_2_ separation. Zhang et al. prepared Pd/ceramic/Ti-Al alloy composite membranes by electroless plating and the composite inorganic membranes showed high stability after three heat cycles.

This Research Topic discusses membrane fouling, engineering antifouling membranes, and high performance FO membranes for water treatment, as well as the development of gas separation membranes. In the future, membrane technology will continue to play a vital role in addressing global environmental challenges, such as water scarcity, climate change, and energy shortages. Engineering new high performance membranes with targeted applications, and understanding the limiting factors and their mechanisms in membrane separation are two key research directions that should be paid more attention to in future research.

## Author Contributions

SZ and LS contributed to the writing of this editorial. Both authors contributed to the article and approved the submitted version.

## Conflict of Interest

The authors declare that the research was conducted in the absence of any commercial or financial relationships that could be construed as a potential conflict of interest.
